# Supplementary material for: Chatbot-based serious games: A useful tool for training medical students? A randomized controlled trial
Source: PLoS One. 2023 Mar 13;18(3):e0278673. doi: 10.1371/journal.pone.0278673 (PMC10010502; doi:10.1371/journal.pone.0278673)
Supplement: S1 Table — (DOCX) [file pone.0278673.s001.docx]

**Supplementary table 1 –** Introductory message upon logging in

| Chat Progress is a study that offers a series of serious games to help teach you pulmonology. The main objective is to evaluate the impact of serious games with a chatbot format on your teaching. All the characters involved are fictional and the scenarios made up. Any resemblance to actual persons, living or dead, or actual events is purely coincidental. We are the evaluation point of the trial, you are allowed to not participate. Your account is purely personal and must not be shared with another student, at the risk of modifying our results.  A satisfaction survey will be sent to you before your end-of-course exam. Your constructive criticism and commentaries will help up improve this new teaching tool.  Enjoy! |
| --- |
