# Supplementary material for: Chatbot-based serious games: A useful tool for training medical students? A randomized controlled trial
Source: PLoS One. 2023 Mar 13;18(3):e0278673. doi: 10.1371/journal.pone.0278673 (PMC10010502; doi:10.1371/journal.pone.0278673)
Supplement: S2 Table — (DOCX) [file pone.0278673.s002.docx]

**Supplementary table 2 –** Satisfaction survey

| 1. Email address 2. What do you think of this game format, of the teaching method?   . Useless  . Average  . Good  . Great   1. Which game did you like the most and why?   Open question   1. Which game did you like the least and why?   Open question     1. Did you make anyone else try it out?   . Yes  . No   1. Did you get game overs voluntarily?   . Yes  . No   1. If you successfully made it through a game, did you ever play the game again?   . Yes  . No   1. Was this format useful for learning concepts?   . Yes  . No   1. Was this format useful for reviewing concepts?   . Yes  . No   1. Do you think the games fit with your training?   . Useless  . Average  . Good  . Great   1. The games’ level of difficulty   . Very hard  . Hard  . Accessible  . Easy  . Very easy   1. The games’ duration   . Too long  . Just right  . Too short   1. Did you enjoy playing the games?   . No  . Average  . Good  . Great   1. Would you like to have access to such a gaming platform for all your teaching modules, with a big variety of games?   . Yes  . No   1. Any advice to improve our games?   Open question |
| --- |
