# Supplementary material for: Chatbot-based serious games: A useful tool for training medical students? A randomized controlled trial
Source: PLoS One. 2023 Mar 13;18(3):e0278673. doi: 10.1371/journal.pone.0278673 (PMC10010502; doi:10.1371/journal.pone.0278673)
Supplement: S3 Table — (DOCX) [file pone.0278673.s003.docx]

**Supplementary table 3 –** Responses to question 15 of the satisfaction survey

| Adding complementary explanation even when the question is right to avoid doing voluntary GO to get one (5)   - More open questions, rather than just MCQs (2) - More games (2) - Harder games (4) - More targeted questions (1) - Use a more formal language (2) - Add more references to the pulmonology text book (1) - Take home messages at the end of a game (3) - Be able to zoom on the pictures (1) - Send reminder emails weekly to use the website (2)   (X): number of students recommending such a change. |
| --- |
