# Supplementary figures and images for: Chatbot-based serious games: A useful tool for training medical students? A randomized controlled trial
Source: PLoS One. 2023 Mar 13;18(3):e0278673. doi: 10.1371/journal.pone.0278673 (PMC10010502; doi:10.1371/journal.pone.0278673)

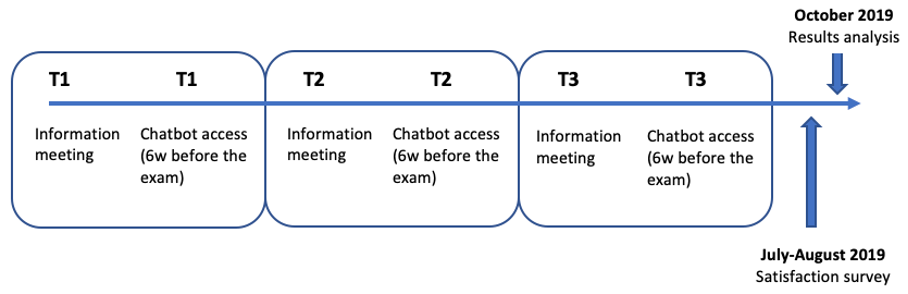

Supplement: S1 Fig — 6w: six weeks. (TIF) [file pone.0278673.s004.tif]

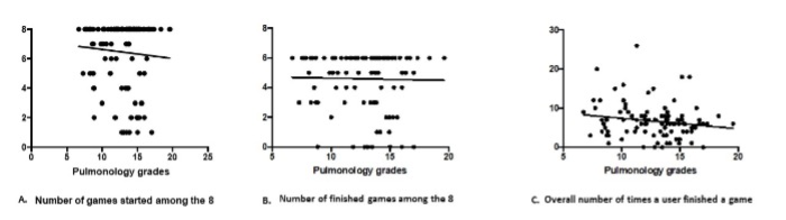

Supplement: S2 Fig — (TIF) [file pone.0278673.s005.tif]
