# Supplementary material for: Chatbot-based serious games: A useful tool for training medical students? A randomized controlled trial
Source: PLoS One. 2023 Mar 13;18(3):e0278673. doi: 10.1371/journal.pone.0278673 (PMC10010502; doi:10.1371/journal.pone.0278673)
Supplement: Attachment — Submitted filename: Response_to_Reviewers.docx [file pone.0278673.s007.docx]

**Response to Reviewers**

**General comments from Editor**

1. **In abstract "The primary aim was to demonstrate an increase in..", "Secondary aims were to demonstrate an increase in scores..". Authors are encouraged to replace "demonstrate" by another term, such as evaluate or assess, and change the rest of the sentence accordingly.**

*🡪 Answer: Changes to the paper were made accordingly, by replacing “demonstrate” by “evaluate”.*

2. **Authors are encouraged to further discuss the low response rate following reviewer's comments**

*🡪 Answer: with the help of Reviewer #2’s comment, we’ve made the necessary changes and added : “This is also why a second blast, a few weeks later, was not sent, although a pre-notification or a subsequent reminder after the initial survey would have increased the response rate (18). An incentive was also not considered, although it was also shown to be linked to an increased response rate, with the budget entirely dedicated to the development of the chatbot and the student’s incentive to use it.*

**Reviewers' comments:**

**Reviewer #2:**

**If I may suggest, perhaps in your limitation "The relatively weak adherence of students to the trial and the survey." you need to add another point where you only did sent out the survey once and no particular strategy was implemented to encourage students to answer the questionnaire, then suggest what strategy can be used to enhance response for future research.**

**Perhaps you can write something as "We only blast the survey once without any reminder or incentive provided. Hence, at least a subsequent reminder is required after the initial survey sent or incentive provided may increase the response rate [XX]. However, due to [your reasons] abstain us from doing such strategies."**

**Reference: Sammut, D. R., Griscti, D. O., & Norman, P. I. J. (2021). Strategies to improve response rates to web surveys: A literature review. International Journal of Nursing Studies, 123, 104058. doi:10.1016/j.ijnurstu.2021.104058**

*🡪 Answer: Thank you very much for all your very interesting comments thus far. We have made the necessary changes, by adding: This is also why a second blast, a few weeks later, was not sent, although a pre-notification or a subsequent reminder after the initial survey would have increased the response rate (18). An incentive was also not considered, although it was also shown to be linked to an increased response rate, with the budget entirely dedicated to the development of the chatbot and the student’s incentive to use it.”*
